# Conference Calendar

**DOI:** 10.1002/cfg.388

**Published:** 2004-03


**Comparative and Functional Genomics**
*Comp Funct Genom* 2004; **5**: 206–208.
Published online in Wiley InterScience (www.interscience.wiley.com). **DOI:** 10.1002/cfg.388

### Conference Calendar

July–September 2004

### Animal models

Comparative Biology and Interactions of Wild and Farmed Fish, 19–23 July
**http://www.easonline.org/agenda/en/description.asp?id=241**

Zebrafish Development and Genetics Meeting, 29 July–2 August
**http://zfin.org/zf_info/news/mtgs.html**

CSHL — Mouse Molecular Genetics, 1–5 September
**http://meetings.cshl.org/2004/2004mouse.htm**

SGM — 155th Meeting of the Society for General Microbiology, 6–9 September
**http://www.sgm.ac.uk/meetings/MTGPAGES/Tcd.cfm**

ISAG — 29th International Conference on Animal Genetics, 11–16 September
**http://www2.kobe-u.ac.jp/%7Eisag2004/**

### Bacteria

GRC — Microbial Toxins and Pathogenicity, 18–23 July
**http://www.grc.uri.edu/programs/2004/micrtox.htm**

CSHL — Molecular Genetics of Bacteria and Phages, 24–29 August
**http://meetings.cshl.org/2004/2004phage.htm**

ASM — 5th International Conference on Extremophiles, 19–23 September
**http://www.asm.org/Meetings/index.asp?bid=19177**

### Bioinformatics

SIAM — Conference on the Life Sciences, 11–14 July
**http://www.siam.org/meetings/ls04/**

4th International Conference on Bioinformatics of Genome Regulation and Structure, 25–30 July
**http://www.bionet.nsc.ru/meeting/bgrs2004/index.html**

ISCB — 12th Intelligent Systems for Molecular Biology (ISMB)/3rd Conference on Computational Biology (ECCB)/Genes, Proteins and Computers VIII, 31 July–1 August
**http://www.iscb.org/ismbeccb2004/**

Computational Systems Bioinformatics Conference, 16–20 August
**http://conferences.computer.org/bioinformatics/**

### Evolution/comparative genomics

Structural Approaches to Sequence Evolution: Molecules, Networks, Populations, 5–10 July
**http://www.mpipks-dresden.mpg.de/~strapp04/**

### General

GRC — Chromatin Structure and Function, 4–9 July
**http://www.grc.uri.edu/programs/2004/chromat.htm**

BS — BioScience 2004, 18–22 July
**http://www.bioscience2004.org/**

GRC — Mitochondria and Chloroplasts, 25–30 July
**http://www.grc.uri.edu/programs/2004/mitochon.htm**

GRC — Macromolecular Organization and Cell Function, 15–20 August
**http://www.grc.uri.edu/programs/2004/macromol.htm**

15th International Chromosome Conference, 5–10 September
**http://www.the-conference.com/2004/iccxv/index.php**

Copyright © 2004 John Wiley & Sons, Ltd.

### Pathways and metabolomics

GRC — Enzymes, Co-enzymes and Metabolic Pathways, 18–23 July
**http://www.grc.uri.edu/04sched.htm**

### Pharmacogenomics

IBC — Drug Discovery Technology, 8–13 August
**http://www.drugdisc.com/section.asp**

Society for Biomolecular Screening Annual Conference, 11–15 September
**http://www.sbsonline.org/**

### Plants

15th International Conference on *Arabidopsis* Research, 11–14 July
**http://www.arabidopsis2004.de/**

GRC — Plant Molecular Biology, 18–23 July
**http://www.grc.uri.edu/04sched.htm**

ASPB — Plant Biology 2004, 24–28 July
**http://www.aspb.org/meetings/pb-2004/**

14th Congress of the Federation of European Societies of Plant Biology, 23–27 August
**http://www.zfr-pan.krakow.pl/konf/#**

Plant GEMS 3, 22–25 September
**http://plant-gems.org/plant_gems/index.html**

### Proteomics

18th Symposium of the Protein Society: Protein Structure, Function and Disease, 14–18 August
**http://www.faseb.org/meetings/protein04/default.htm**

CHI — Protein Biomarkers, 30 August–1 September
**http://www.healthtech.com/conference-fall04.asp**

3rd International and 28th European Peptide Symposium, 5–10 September
**http://kenes.com/28eps/index.html**

### Structural genomics

GRC — Diffraction Methods in Structural Biology, 11–16 July
**http://www.grc.uri.edu/programs/2004/diffrac.htm**

5th International Conference on Biological Physics, 23–27 August
**http://fy.chalmers.se/icbp2004/**

IAPSAP — 15th Meeting on Methods in Protein Structure Analysis, 29 August–2 September
**http://depts.washington.edu/biowww/mpsa2004/**

### Systems biology

BTK — Developing Concepts for Systems Biology, 3–6 September
**http://mudshark.brookes.ac.uk/BTK/**

### Transcriptomics

GRC — The Biology of Post-transcriptional Gene Regulation, 8–13 August
**http://www.grc.uri.edu/programs/2004/posttran.htm**

CHI — Microarray Data Analysis, 18–20 August
**http://www.healthtech.com/conference-fall04.asp**

CSHL — Dynamic Organization of Nuclear Function, 29 September–3 October
**http://meetings.cshl.org/2004/2004nucleus.htm**

### Yeasts and fungi

GSA — Yeast Genetics and Molecular Biology Meeting, 27 July–1 August
**http://genetics.faseb.org/genetics/yeast/**

### Biotech industry

BIOPHEX 2004: Post-discovery through Commercialization, 28–30 September
**http://www.biophex.com/**

Copyright © 2004 John Wiley & Sons, Ltd.

### Key

ASM — American Society for Microbiology: **http://www.asm.org**

ASPB — American Society of Plant Biologists: **http://www.aspb.org/**

BS — Biochemical Society: **http://www.biochemistry.org/**

BTK — BioThermoKinetics Study Group: **http://dbk.ch.umist.ac.uk/btk/home.htm**

CHI — Cambridge Healthtech Institute: **http://www.healthtech.com**

CSHL — Cold Spring Harbor Laboratories: **http://meetings.cshl.org/index.htm**

GRC — Gordon Research Conferences: **http://www.grc.uri.edu/**

GSA — Genetics Society of America: **http://www.genetics-gsa.org/**

IAPSAP — International Association for Protein Structure Analysis and Proteomics: **http://www.iapsap.bnl.gov/**

IBC — IBC Conferences: **http://www.ibc-lifesci.com**

ISAG — International Society for Animal Genetics: **http://www.isag.org.uk/**

ISCB — International Society for Computational Biology: **http://www.iscb.org**

SGM — Society for General Microbiology: **http://www.sgm.ac.uk**

SIAM — Society for Industrial and Applied Mathematics: **http://www.siam.org/**

The Conference Calendar is a listing of forthcoming conferences of interest to our readership. We provide the title, dates and web address of each conference, to enable readers to find out more information. Inclusion does not imply endorsement by the journal. If you know of a relevant conference, please contact the Managing Editor.

Copyright © 2004 John Wiley & Sons, Ltd. 

